# Premature ventricular complexes and risk of atrial fibrillation and stroke in patients without structural heart disease

**DOI:** 10.1136/heartjnl-2024-325322

**Published:** 2025-06-23

**Authors:** Robin Bouleau, Natalie Glaser, Martin Jonsson, Raffaele Scorza

**Affiliations:** 1 Department of Cardiology, Södersjukhuset, Stockholm, Sweden; 2 Department of Clinical Science and Education, Södersjukhuset, Karolinska Institutet, Stockholm, Sweden; 3 Department of Molecular Medicine and Surgery, Karolinska Institutet, Stockholm, Sweden

**Keywords:** Atrial Fibrillation, STROKE, Epidemiology, Ventricular Premature Complexes

## Abstract

**Background and objective:**

Prior studies have suggested that patients with premature ventricular complexes (PVCs) may have an increased risk for atrial fibrillation (AF) and stroke. It is unclear whether frequent PVCs are linked to an increased risk of AF and stroke in patients where structural heart disease (SHD) has been excluded. We aimed to study if PVCs increase the risk of AF or stroke in patients without SHD.

**Methods:**

In this retrospective observational cohort study, we included patients who received a PVC diagnosis at three major hospitals in Stockholm, Sweden. The patients had no history of heart disease, normal results at stress test and echocardiography, and no previous diagnosis of AF or stroke. For each case, four matched controls were obtained from the general population. We used inverse probability weighting (IPW) to control for differences in baseline characteristics.

**Results:**

A total of 751 PVC patients and 3041 controls were included. The median age was 59 years, and 2239 (59%) were women. The median follow-up time was 5.2 years. There was a higher risk of AF among patients in the PVC group compared with the control group in the unadjusted analysis (HR 2.08, 95% CI 1.35 to 3.20, p=0.0009). After IPW, there was no significant difference in the risk of AF (HR 1.44, 95% CI 0.88 to 2.37) or stroke (HR 1.32, 95% CI 0.81 to 2.14) between the PVC group and the control group.

**Conclusion:**

In patients with PVCs but without SHD, there was no increased risk of AF or stroke compared with controls from the general population after adjusting for known confounders. However, PVCs were associated with AF in the crude cohort, suggesting that PVCs may be a clinical marker for AF.

**WHAT IS ALREADY KNOWN ON THIS TOPIC**

- Previous studies have shown an association between premature ventricular complexes (PVCs) and atrial fibrillation (AF) and stroke. Whether this also applies to patients without structural heart disease is unclear.

**WHAT THIS STUDY ADDS**

- After adjusting for known confounders, PVC patients without structural heart disease did not have a significantly higher risk of AF or stroke than matched controls from the general population.

**HOW THIS STUDY MIGHT AFFECT RESEARCH, PRACTICE OR POLICY**

- While the results of this study do not support the idea that PVCs lead to AF and stroke, PVCs may serve as a marker for AF. Measures to reduce the risk of AF, such as lifestyle interventions, might be warranted in these patients.

## Introduction

 Premature ventricular complexes (PVCs) are a common form of arrhythmia and can be found in most patients undergoing long-term ambulatory monitoring.^1^ They are present both in those with and without structural heart disease (SHD)^2 3^ and can be asymptomatic or present with symptoms such as palpitations, dyspnoea, presyncope and fatigue.^1^ PVCs are a known predictor of poor outcome in patients with pre-existing heart disease,^4 5^ but previous studies have found contradictory results in patients without SHD. Some studies have shown a favourable prognosis for PVC patients who were free from SHD,^6 7^ whereas others found an association between PVCs and cardiac diseases such as cardiomyopathy, ischaemic heart disease, malignant arrhythmias and sudden cardiac death.^8 9^ Previous studies have suggested that PVCs might also be a risk factor for ischaemic stroke, but the evidence is limited and not conclusive.^10^,^13^ Furthermore, it has been suggested that elevated PVC counts on 24-hour ECG predict incident atrial fibrillation (AF)^14^ and that PVCs increase the risk of new-onset AF,^15 16^ providing a possible pathogenetic way leading from PVCs to ischaemic stroke through AF.

Prior studies investigating PVCs and the risk of AF or stroke often included patients with heart disease or excluded them without the use of imaging and functional diagnostic methods to detect occult SHD. Therefore, we conducted a study to evaluate the association between PVCs and the risk of AF and stroke in patients where SHD had been ruled out through echocardiography and stress test.

## Methods

### Study design

This retrospective observational cohort study was conducted in accordance with the STROBE (Strengthening the Reporting of Observational Studies in Epidemiology) guidelines.^17^

### Setting

We included patients who received a PVC diagnosis at cardiology centres at three major hospitals in Stockholm, Sweden, between 1 January 2010 and 31 December 2016. The hospitals are secondary centres and cover approximately 15% of the Swedish population. The patients were referred to the cardiology centres from primary care, emergency care or after initial evaluation by a cardiologist due to various reasons. Follow-up for AF, transitory ischaemic attack (TIA), stroke and survival ended on 31 December 2018.

### Study population

Through a review of medical records, we identified all patients who received a PVC diagnosis at three cardiology centres in Stockholm, Sweden, between 2010 and 2016. The International Classification of Diseases, Tenth Revision (ICD-10) code to define PVCs was I49.3. Patients with PVCs as both primary and secondary diagnoses were identified. All ECGs were reviewed by an experienced cardiologist (RS) to confirm the diagnosis. Only patients who had undergone continuous ECG recording (at least 24-hour ambulatory monitoring or telemetry), who had a normal echocardiogram and a normal stress test were included. Normal results on echocardiography were defined as left ventricular ejection fraction equal to or higher than 55%, absence of more than mild valvular disease, normal ventricular dimensions and normal left ventricular wall thickness. A normal stress test result was defined as the absence of electrocardiographic findings indicating coronary artery disease or stress-induced ventricular arrhythmia. Both echocardiography and stress tests were assessed by experienced clinical physiologists. In a few cases, supplementary evaluation with MRI, myocardial scintigraphy or coronary angiography was performed at the request of the treating cardiologist to exclude SHD. Patient data was linked to the National Patient Register,^18^ and all patients with a history of previous heart disease (heart failure, previous myocardial infarction, sustained ventricular tachycardia, survived cardiac arrest, moderate or severe valvular heart disease, previous cardiac surgery or percutaneous coronary revascularisation) were excluded. Patients with a prior history of AF, TIA or stroke were also excluded. PVC patients were divided into four groups depending on PVC burden (less than 1000 PVCs/day, 1000–4999 PVCs/day, 5000–9999 PVCs/day and more or equal to 10 000 PVCs/day).

**Figure 1:**
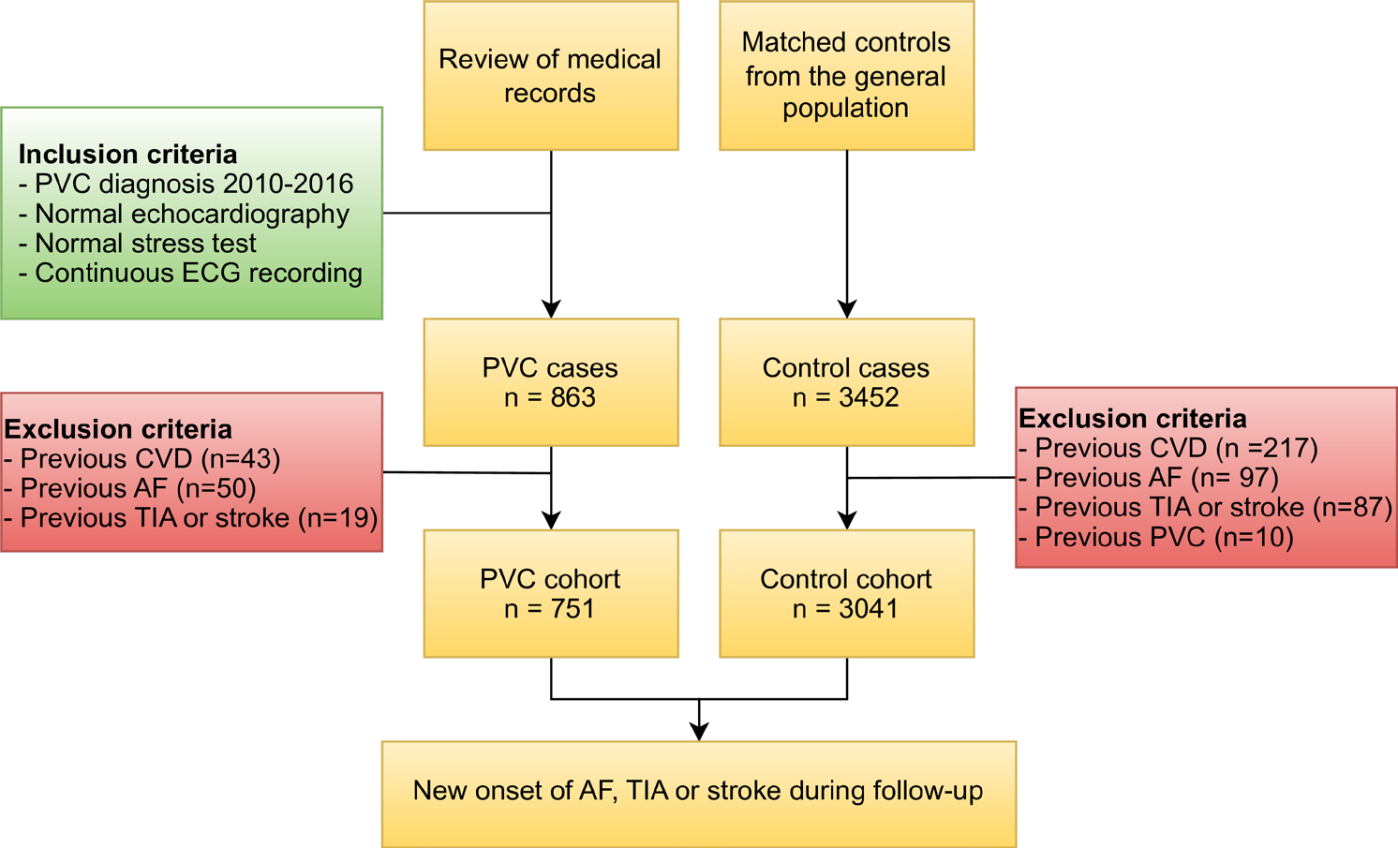
Flow chart of the study selection process. AF, atrial fibrillation; CVD, cardiovascular disease; PVC, premature ventricular complex; TIA, transitory ischaemic attack.

Every PVC patient was linked to four age-matched and sex-matched controls from a random sample of the general Swedish population. The controls were identified by Statistics Sweden and cross-linked to the National Patient Register.^18^ Controls with a prior cardiovascular diagnosis, including PVC, AF, TIA and stroke, were excluded. A flowchart of the inclusion process is shown in figure 1.

### Exposure and outcomes

The exposure was having received a PVC diagnosis (ICD-code I49.3) between 2010 and 2016. The exposure was identified through a review of medical records. Outcomes were new onset of AF (ICD-10-code I48) and a combined endpoint of TIA (ICD-10-code G45.9) and stroke (ICD-10-codes I63, I64, I67, I69.3 and I69.4). The outcomes were identified by linking data from PVC patients and controls to the National Patient Register.^18^

### Data sources

The unique 12-digit personal identification number assigned to all citizens in Sweden^19^ was used to cross-link all PVC patients and controls to the National Patient Register^18^ and the Swedish Prescribed Drugs Register,^20^ both run by the National Board of Health and Welfare. Data from the registries cover in-hospital care and hospital-linked out-patient care in Sweden from 1987 and 2005, respectively. The registries were used to obtain data on outcomes, survival status and baseline characteristics, including prescribed drugs at the time of inclusion. ICD-10 and Anatomical Therapeutic Chemical Classification System codes that defined baseline characteristics are presented in online supplemental table 1.

### Statistical methods

Baseline characteristics are presented as medians and IQRs for continuous variables and as counts and percentages for categorical variables. Differences in baseline characteristics between the PVC group and the control group are presented as standardised mean differences (SMDs). An SMD larger than 0.1 was considered a relevant difference. Time to event was defined as the number of days between inclusion and time of first diagnosis of either AF or TIA/stroke. Cox proportional hazard regression was used to estimate HRs and 95% CIs for the unadjusted analyses. Crude cumulative incidence rates are presented as survival curves. To balance potential confounders, inverse probability weighting (IPW) based on the propensity score was used. Variables used for the weighting were hypertension, diabetes, chronic kidney disease, hyperthyroidism, cerebrovascular disease (other than stroke/TIA), hyperlipidaemia, alcohol dependency and obesity, as well as known prescription of beta-blockers, calcium channel blockers, ACE inhibitors, angiotensin receptor blockers, diuretics, antithrombotic therapy (including antiplatelet agents, heparin, vitamin K antagonists and non-vitamin K oral anticoagulants) and antiarrhythmic drugs. To estimate the weighted cumulative incidence function, we used a proportional subdistribution hazards’ regression model^21^ with death as a competing risk using the cmprskcoxmsm package in R. The method considers both the propensity score weighting and the fact that patients who die during follow-up cannot receive a diagnosis of AF or TIA/stroke in the future. Results are reported as adjusted cumulative incidence functions and HR with 95% CI. Data management and statistical analyses were performed in R V.4.2.2 (R Foundation, Vienna, Austria).

### Missing data

Data was missing for PVC burden in 143 PVC patients (19%). This variable was not used in any of the regression analyses. The remaining variables have no missing data.

### Patient and public involvement

Patients were not involved in the research process of this study.

## Results

### Study population

We included a total study population of 751 PVC patients and 3041 controls. The median age was 59 years (IQR 45–69), and 2239 (59%) were women. The PVC population was more likely to have hypertension (19% vs 11%) and hyperlipidaemia (7% vs 3%). The PVC population was prescribed more beta blockers (47% vs 17%), calcium channel blockers (26% vs 16%), antithrombotic therapy (24% vs 15%) and diuretics (37% vs 27%). Baseline characteristics are presented in table 1. After IPW, baseline characteristics were well-balanced between the groups (online supplemental figure 1). Among the PVC patients, 465 (76%) had more than 1000 PVCs/day and 174 (29%) had equal to or more than 10 000 PVCs/day. In the PVC group, 21 (3%) patients died during follow-up, compared with 130 (4%) persons in the control group.

**Table 1:** Baseline demographics and clinical characteristics of the study population

Variable	PVC group	Controls	SMD
No.	751	3041	
Age, years (median (IQR))	59 (45–69)	59 (45–69)	<0.01
Female sex	445 (59.3)	1794 (59.0)	0.01
Hypertension	142 (18.9)	321 (10.6)	0.24
Diabetes	31 (4.1)	146 (4.8)	0.03
Chronic kidney disease	5 (0.7)	12 (0.4)	0.03
Hyperthyroidism	11 (1.5)	26 (0.9)	0.06
Cerebrovascular disease (other than stroke/TIA)	4 (0.5)	15 (0.5)	0.01
Hyperlipidaemia	50 (6.7)	77 (2.5)	0.20
Alcohol dependency	13 (1.7)	70 (2.3)	0.04
Obesity	20 (2.7)	60 (2.0)	0.05
Beta blockers	356 (47.4)	510 (16.8)	0.69
Calcium channel blockers	192 (25.6)	479 (15.8)	0.24
ACE inhibitors	131 (17.4)	429 (14.1)	0.09
Angiotensin receptor blockers	89 (11.9)	311 (10.2)	0.05
Antithrombotic therapy	181 (24.1)	459 (15.1)	0.23
Diuretics	277 (36.9)	808 (26.6)	0.22
Antiarrhythmic drugs class 1	8 (1.1)	2 (0.1)	0.13
Antiarrhythmic drugs class 3	4 (0.5)	0 (0.0)	0.10

Numbers are No. (%) unless otherwise noted.

PVC, premature ventricular complex; SMD, standardised mean difference; TIA, transitory ischaemic attack.

### Atrial fibrillation

**Figure 2:**
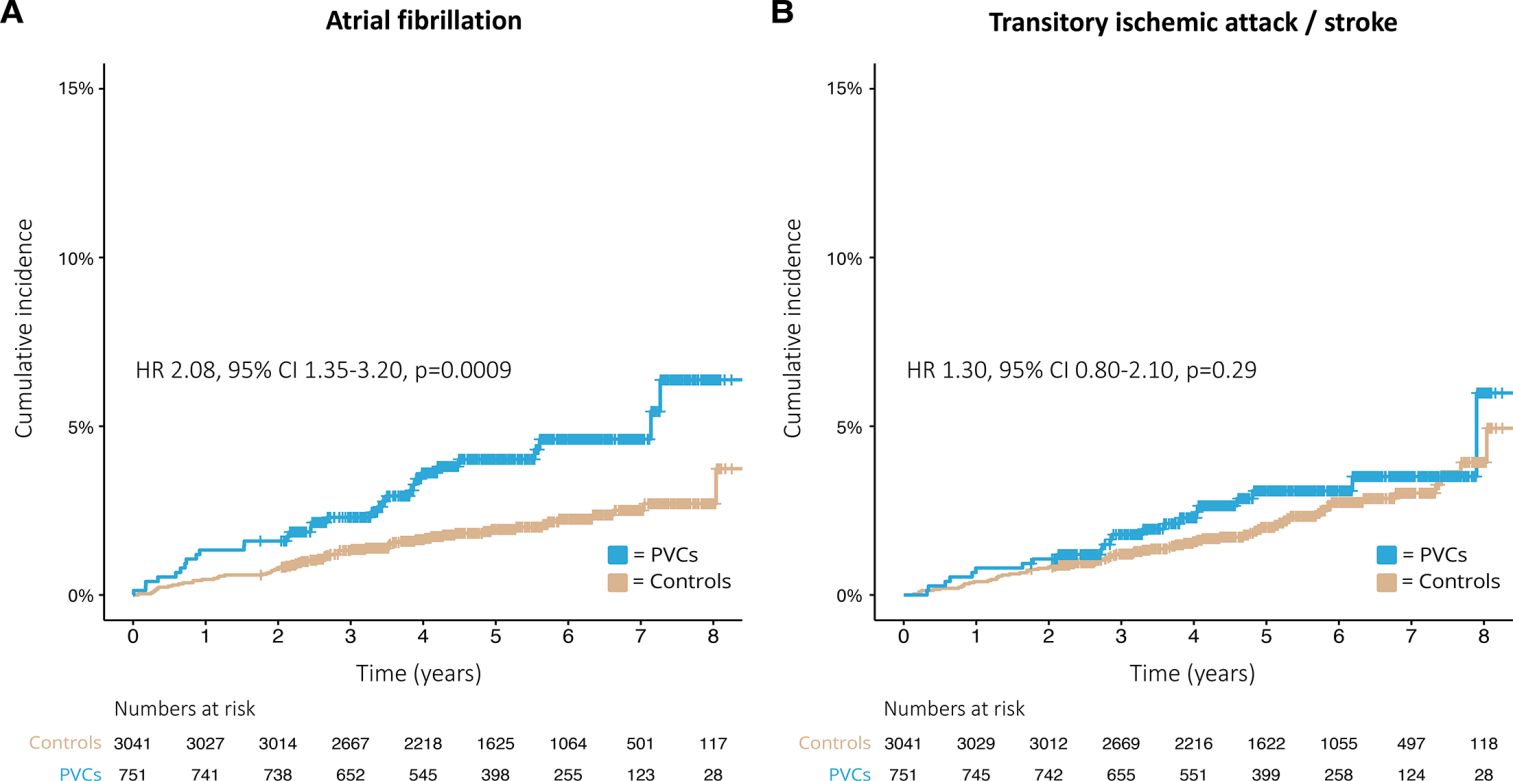
Unadjusted Kaplan-Meier curves showing crude cumulative incidence rates of (A) Atrial fibrillation and (B) Transitory ischaemic attack/stroke in 751 patients with premature ventricular complexes (PVCs) and 3041 controls between 2010 and 2018.

**Figure 3:**
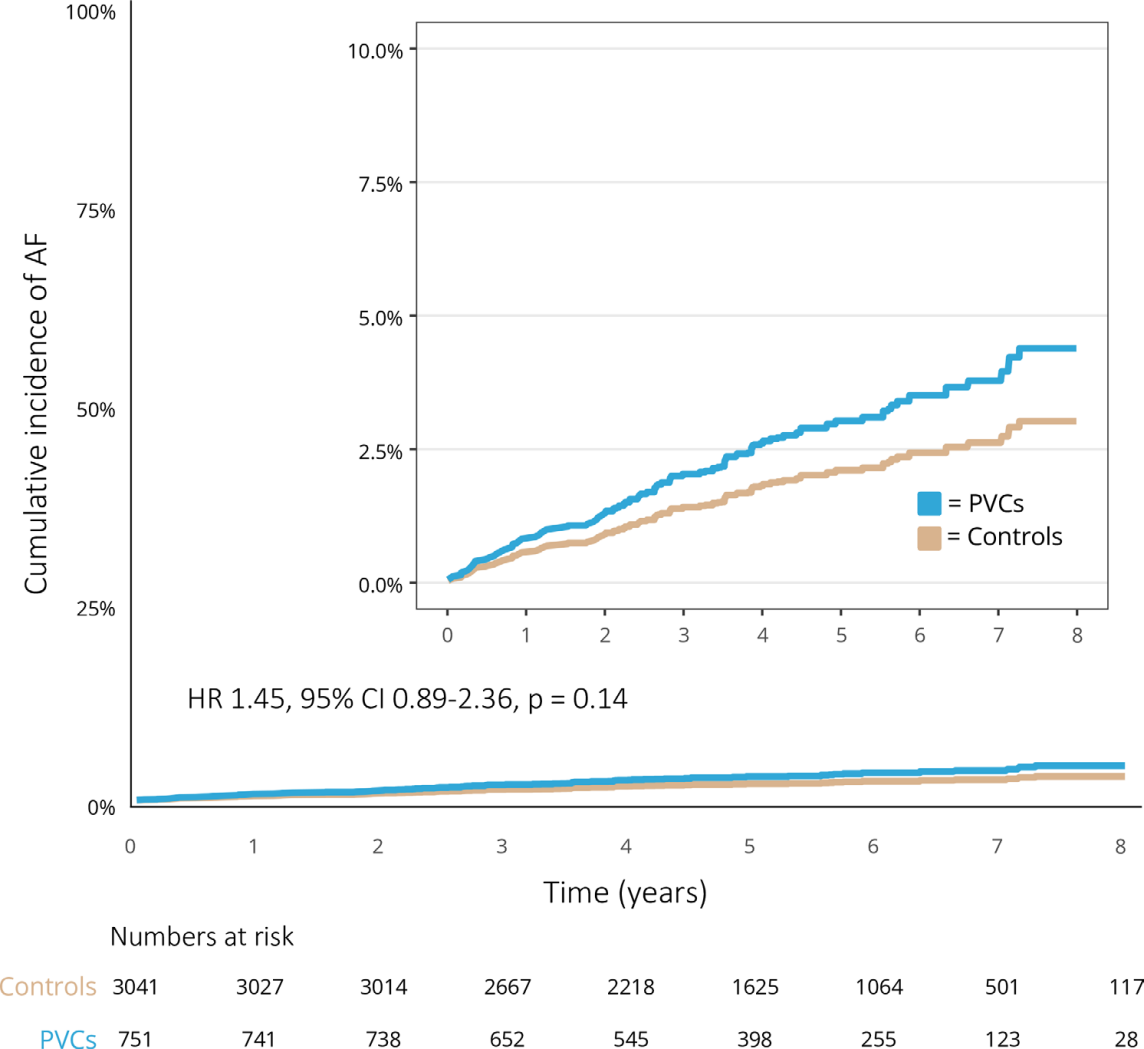
Cumulative incidence of atrial fibrillation in the weighted cohort consisting of 751 patients with premature ventricular complexes (PVCs) and 3041 controls between 2010 and 2018 taking competing risks into account. AF, atrial fibrillation.

During a median follow-up time of 5.2 years (IQR 3.9–6.5), a total of 31 (4.1%) patients in the PVC group and 61 (2.0%) persons in the control group were diagnosed with AF. The unadjusted incidence rate of AF was 8.1 per 1000 person years in the PVC group and 3.9 per 1000 person years in the control group. The crude cumulative incidence of AF in the PVC group at 1 year was 1.3% compared with 0.5% in the control group; at 3 years 2.3% versus 1.4%; and at 5 years 4.0% versus 1.9%. There was a higher risk of AF among patients in the PVC group compared with the control group in the unadjusted analysis (HR 2.08, 95% CI 1.35 to 3.20, p=0.0009). The crude cumulative incidence of AF in the PVC group versus the control group is presented in figure 2. The adjusted cumulative incidence of AF at 1 year was 0.8% in the PVC group compared with 0.6% in the control group; at 3 years 2.1% versus 1.4%; and at 5 years 3.1% versus 2.1%. After IPW, there was no significant difference in risk for AF between the PVC group and the control group (HR 1.45, 95% CI 0.89 to 2.36, p=0.14). Figure 3 shows the adjusted cumulative incidence of AF. Among patients with less than 1000 PVCs per 24 hours, 3 (2.1%) patients received a diagnosis of AF during follow-up, compared with 12 (4.1%) among patients with 1000 to 9999 PVCs per 24 hours and 9 (5.2%) patients in the group with equal to or more than 10 000 PVCs per 24 hours. Due to the low number of outcome events, no further analysis was performed on this data. The prevalence of AF in relation to PVC burden is presented in online supplemental table 3.

### Transitory ischaemic attack and stroke

**Figure 4:**
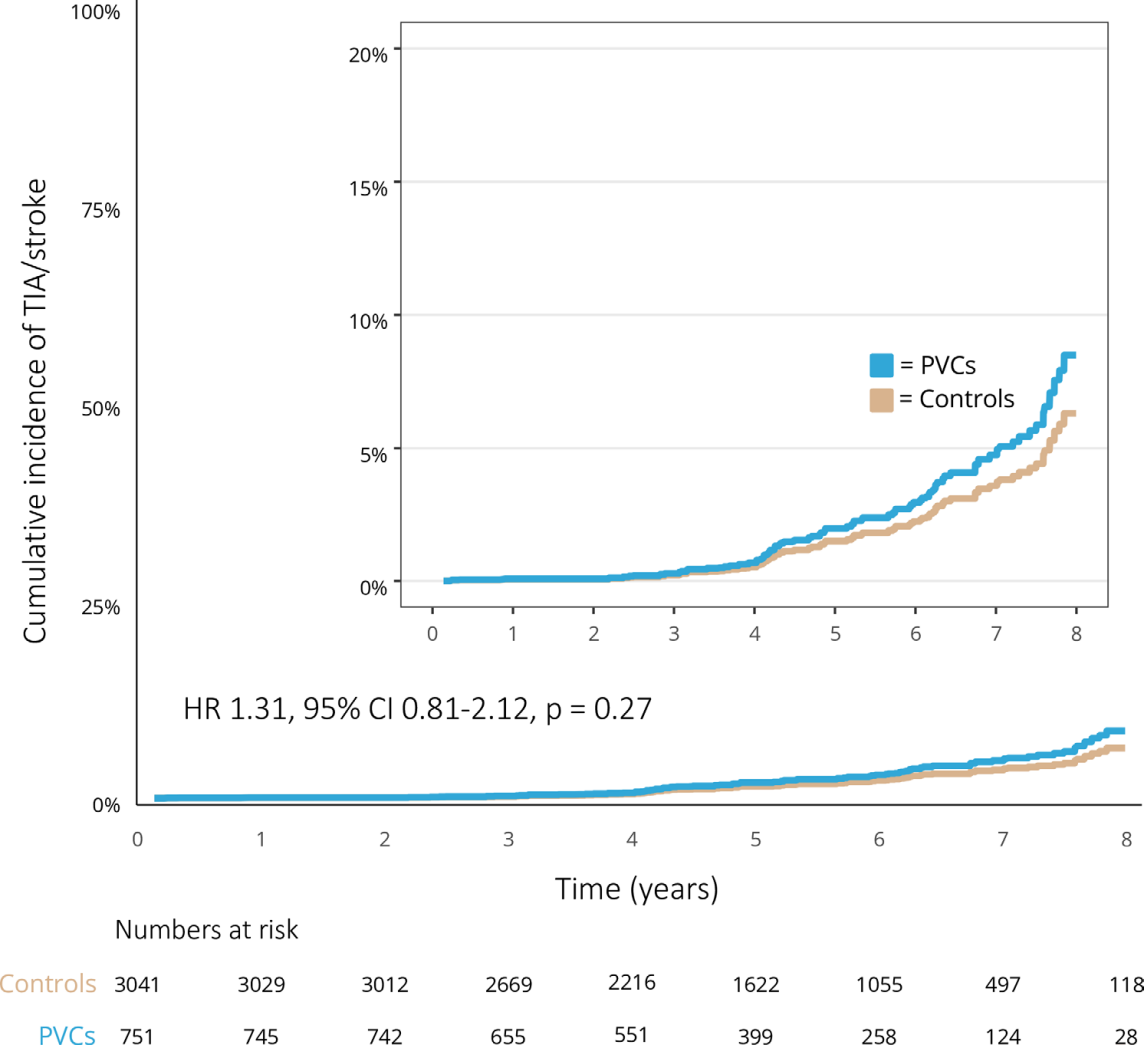
Cumulative incidence of transitory ischaemic attack and stroke in the weighted cohort consisting of 751 patients with premature ventricular complexes (PVCs) and 3041 controls between 2010 and 2018 taking competing risks into account. TIA, transitory ischaemic attack.

During a median follow-up time of 5.2 years (IQR 3.9–6.5), a total of 22 (2.9%) patients in the PVC group and 69 (2.3%) persons in the control group received a diagnosis of TIA or stroke. The unadjusted incidence rate of TIA and stroke was 5.7 per 1000 person years in the PVC group and 4.4 per 1000 person years in the control group. The crude cumulative incidence of TIA and stroke at 1 year was 0.8% compared with 0.4% in the control group; at 3 years 1.8% versus 1.2%; and at 5 years 3.1% versus 2.0%. There was no difference in the risk of TIA and stroke among patients in the PVC group compared with the control group in the unadjusted analysis (HR 1.30, 95% CI 0.80 to 2.10, p=0.29). The crude cumulative incidence of TIA and stroke in the PVC group versus the control group is presented in figure 2. The adjusted cumulative incidence of TIA and stroke at 1 year was 0.08% in the PVC group compared with 0.06% in the control group; at 3 years 0.28% versus 0.21%; and at 5 years 2.0% versus 1.5%. After IPW, there was no significant difference in risk for the combined endpoint of TIA or stroke between the PVC group and the control group (HR 1.31, 95% CI 0.81 to 2.12, p=0.27). Figure 4 shows the adjusted cumulative incidence of TIA and stroke. Among patients with less than 1000 PVCs per 24 hours, 3 (2.1%) patients received a diagnosis of TIA or stroke during follow-up, compared with 13 (4.5%) among patients with 1000 to 9999 PVCs per 24 hours, and 4 (2.3%) patients in the group with equal to or more than 10 000 PVCs per 24 hours. The prevalence of TIA and stroke in relation to PVC burden is presented in online supplemental table 3.

## Discussion

In this retrospective observational cohort study, we found no increased risk for AF or stroke in patients with PVCs without SHD compared with a control group from the general Swedish population. To our knowledge, our study is the first study exploring PVCs and risk of AF and stroke where all PVC patients have undergone a thorough clinical investigation, including echocardiography and stress test, to rule out SHD.

Several studies have previously shown an association between PVCs and AF or stroke.^11^,^1622^ Therefore, it has been speculated that there is a possible causal relationship between PVCs and AF or stroke and that PVC ablation might lower the risk of developing these diseases.^13 15 16 22^ In contrast, others believe that PVCs are more likely to be a marker of some other cardiac pathology that affects the electrical properties of the heart.^14^ However, previous studies either included patients with SHD (eg, heart failure, ischaemic heart disease or valvular heart disease) or excluded them without the use of imaging and functional testing such as echocardiography and stress test, to find patients with previously unknown SHD.

In a meta-analysis by Rujirachun *et al*, including six cohort studies and 9 622 088 individuals, the authors showed a 1.9-fold increased risk of AF in patients with PVCs compared with patients without PVCs.^23^ However, four of the six studies included patients with previous cardiovascular disease, and the authors concluded that it is possible that PVCs simply are a marker for cardiovascular morbidity that, in turn, leads to AF and that further studies are needed to investigate a possible causal relationship between PVCs and AF.^23^ Another meta-analysis by the same authors investigated the association between PVCs and stroke, including four observational studies and 42 677 participants.^10^ This study found that PVCs were associated with an increased risk of ischaemic stroke but stated that the association could be explained by a higher prevalence of cardiovascular disease in the PVC patients.^10^

While PVCs are known to be associated with SHD, the actual cause is often idiopathic.^1^ While some PVCs originate in structural cardiac defects, such as scar tissue after myocardial infarction,^24^ others may arise due to more subtle changes in the cardiomyocyte. For example, PVCs originating in the right ventricular outflow tract have been speculated to be caused by deviations during embryonic development, causing increased automaticity in these cells.^25^ By excluding underlying SHD in our PVC population, we aimed to select a subgroup of patients with PVCs of another pathophysiological origin than structural abnormalities, which could explain the negative finding in our study compared with prior studies.

It is well established that a high burden of PVCs is associated with a reversible form of heart failure,^26 27^ and several of the pathophysiological changes that come with heart failure, including pressure and volume overload, interstitial fibrosis, altered atrial refractory properties and heterogeneity of conduction, can predispose to AF.^28^ Many of the factors that increase the risk of developing heart failure, such as hypertension, valvular disease and ischaemic heart disease, also increase the risk of AF.^28^ In a similar way, stroke shares both pathophysiological mechanisms as well as many risk factors with ischaemic heart disease,^29^ which in turn is a known risk factor for PVCs.^2 5^

The use of antiarrhythmic drugs class one was higher in the PVC group, which may have led to an underestimate of AF in this population. However, the total number of patients on these medications was low (n=14), so the difference should be interpreted cautiously. Furthermore, the prescription of antithrombotic therapy (including vitamin-K-antagonists, heparins, platelet inhibitors, thrombolytics, direct thrombin inhibitors and factor Xa-inhibitors) was higher in the PVC group. The subtype of antithrombotic therapy prescribed can be seen in online supplemental table 4. A majority (49%) of the prescriptions consisted of aspirin, followed by low molecular weight heparin (44%). While we lack information about the reasons for the prescriptions, it is reasonable to believe that this may have affected the outcome of TIA and stroke in favour of the PVC group.

The results of our study add to the belief that it is not PVCs in themselves that increase the risk of AF and stroke but the underlying heart disease that PVCs sometimes represent. When dividing the PVC population into groups based on PVC burden, there was a trend towards a higher incidence of AF in patients with more PVCs, which could suggest that patients with a high number of PVCs are at increased risk of developing AF. While the number of events per group was low, and these unadjusted results should be interpreted with caution, it is possible that we would have seen a significant difference between the groups with a larger study population, highlighting the need for further studies with more patients and longer follow-up.

### Strengths and limitations

The clinical and demographic characteristics of the PVC patients in our study are well-defined, which increases internal validity. Sweden’s high quality national registers ensure that we have comprehensive data on outcomes, comorbidities and medications from both in-patient and hospital-linked out-patient care. However, data from primary care is missing, which means that our study may lack data on diagnoses commonly managed in primary care settings, such as hypertension and obesity. We also have no information on whether the PVC patients had previously been diagnosed with PVCs or evaluated for palpitations in primary care, which could explain the higher rate of prescription of beta-blockers in the PVC group. There may also be patients and controls who have been diagnosed with AF in primary care, but this should occur equally in both groups and is therefore not likely to affect our results. Furthermore, we did not have information about specific echocardiographic parameters.

While the patients in the PVC group were included at three cardiology centres in the Stockholm area, the controls are a sample of the general Swedish population. Geographical differences in search patterns and accessibility to healthcare may have influenced the results. However, all patients who received a PVC diagnosis at the included hospitals during the study period were assessed for eligibility, which increases the external validity. All PVC patients have been followed by a cardiologist, at least at the time of inclusion. While we do not have information on the frequency or type of monitoring the patients underwent after inclusion, it is reasonable to believe that they were offered a more intense follow-up than the controls, which could have increased the likelihood of finding subclinical AF in this group. Also, the control group was not as well examined as the PVC group, and thus, it is possible that controls with unknown underlying SHD or subclinical PVCs were included. Since we do not have information on monitoring after inclusion, such as repeated Holter monitoring, we lack information on the progression of PVC burden.

The assessment of the PVC patients was carried out by the treating cardiologist, and all have undergone basic investigation with ECG and echocardiography in accordance with current guidelines at the time of inclusion.^30^ However, only a few have undergone further investigation with contrast-enhanced cardiac MRI, which could mean that some patients with structural abnormalities missed on echocardiography may have been included.

To investigate new-onset AF and stroke, patients and controls with a previous history of these diagnoses were excluded. The exclusion of these patients may have caused an underestimation of the true number of patients who had AF or stroke/TIA during follow-up.

To our knowledge, the present study is the largest to explore PVCs and the risk of AF and stroke, with all patients thoroughly investigated to rule out SHD. However, it is possible that we would have detected a significant difference between the groups if we had had a larger study population and more events. Finally, data for the outcome measures were only available until 2018, which limited the follow-up time of the study.

## Conclusion

In this observational retrospective analysis of patients with PVCs, where SHD had been excluded through echocardiography and stress test, there was no difference in the risk of AF or stroke compared with controls from the general population after adjusting for known confounders. While these results do not support the idea that PVCs lead to AF or stroke, there was a significant association between PVCs and AF in the crude cohort, which could imply that PVCs may serve as a clinical marker for AF. More studies are needed to fully comprehend potential risks and their mechanisms, especially in patients with frequent PVCs.

## Supplementary material

10.1136/heartjnl-2024-325322 online supplemental file 1

10.1136/heartjnl-2024-325322 online supplemental file 3

## Data Availability

Data may be obtained from a third party and are not publicly available.
