# Supplementary material for: Premature ventricular complexes and risk of atrial fibrillation and stroke in patients without structural heart disease
Source: Heart. 2025 Jun 23;112(1):e325322. doi: 10.1136/heartjnl-2024-325322 (PMC12703231; doi:10.1136/heartjnl-2024-325322)

PVCs were not associated with an increased risk of AF or stroke in patients without structural heart disease

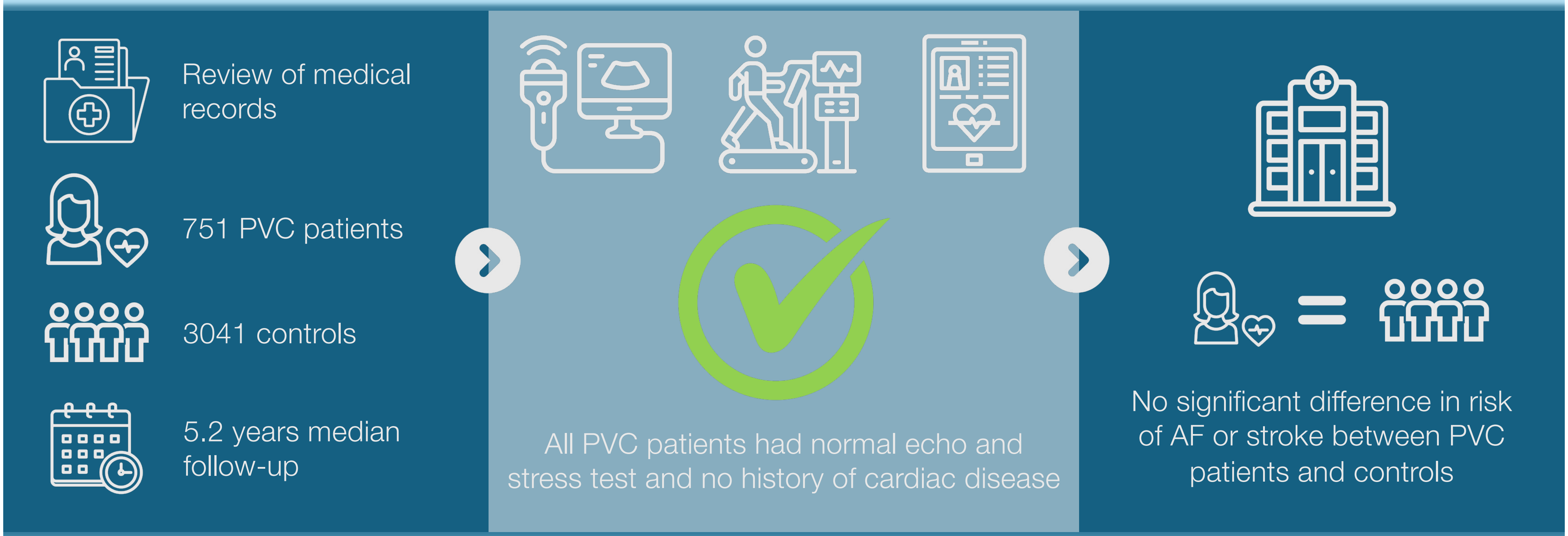

Supplement: online supplemental file 1 [file heartjnl-112-1-s001.pdf]
