# Supplementary material for: Premature ventricular complexes and risk of atrial fibrillation and stroke in patients without structural heart disease
Source: Heart. 2025 Jun 23;112(1):e325322. doi: 10.1136/heartjnl-2024-325322 (PMC12703231; doi:10.1136/heartjnl-2024-325322)
Supplement: online supplemental file 3 [file heartjnl-112-1-s002.pdf]

# SUPPLEMENTAL MATERIAL

Premature Ventricular Complexes and Risk of Atrial Fibrillation and Stroke in Patients Without Structural Heart Disease

Corresponding Author:
Robin Bouleau, MD
Department of Clinical Science and Education
Karolinska Institutet, Södersjukhuset
Sjukhusbacken 10
118 83 Stockholm, Sweden
+46 08-123 630 49 | +46 73-906 94 48
Robin.Bouleau@ki.se

## Table of content

1. Table S1. The International Classification of Diseases, Tenth Revision (ICD-10) codes used to define baseline characteristics.
2. Table S2. Anatomical Therapeutic Chemical Classification System (ATC) codes used to define medications in baseline characteristics.
3. Table S3. New onset of atrial fibrillation and TIA/stroke during follow-up in relation to PVC burden.
4. Table S4. Subtypes of prescribed antithrombotic therapy.
5. Figure S1. Balance of baseline characteristics before and after inverse probability weighting (IPW).

Table S1.

Table S1. The International Classification of Diseases, Tenth Revision (ICD-10) code to define baseline characteristics.

| Variable | ICD-10 code |
|---|---|
| Alcohol dependency | K70, F10 |
| Hypertension | I10 |
| Diabetes | E10-E14 |
| Chronic kidney disease | N18 |
| Hyperthyroidism | E05 |
| Cerebrovascular disease (other than stroke/TIA) | I65, I66, I68, I69.0, I69.1, I69.2, I69.8 |
| Hyperlipidemia | E78 |
| Obesity | E66 |

Table S2.

**Table S2. Anatomical Therapeutic Chemical Classification System (ATC) codes used to define medications.**

| Medication | ATC code |
|---|---|
| Beta-blockers | C07 |
| Calcium channel blockers | C08 |
| ACE-inhibitors | C09A, C09B |
| Angiotensin receptor blockers | C09C, C09D |
| Antithrombotic therapy | B01A |
| Diuretics | C03, C02D, C02L |
| Antiarrhythmic drugs class 1 | C01BA, C01BB, C01BC |
| Antiarrhythmic drugs class 3 | C01BD |
| Digitalis | C01AA |

Table S3.

| Table S3. New onset of atrial fibrillation and TIA/stroke during follow-up in relation to PVC burden | | | |
|---|---|---|---|
| PVCs/24h | No. (%) | Atrial fibrillation | TIA/stroke |
| <1000 | 143 (24) | 3 (2.1%) | 3 (2.1%) |
| 1000-4999 | 194 (32) | 8 (4.1.%) | 8 (4.1%) |
| 5000-9999 | 97 (16) | 4 (4.1%) | 5 (5.2%) |
| ≥10.000 | 174 (29) | 9 (5.2%) | 4 (2.3%) |

TIA = transitory ischemic attack, PVC = premature ventricular complex.

Table S4.

Table S4. Anatomical Therapeutic Chemical Classification System (ATC) codes of subtypes of prescribed antithrombotic therapy

| ATC code | PVC group (n) | Control group (n) |
|---|---|---|
| B01AA03 (Warfarin) | 4 | 34 |
| B01AB01 (Heparin) | 1 | 4 |
| B01AB04 (Dalteparin) | 50 | 99 |
| B01AB05 (Enoxaparin) | 17 | 27 |
| B01AB10 (Tinzaparin) | 12 | 39 |
| B01AC04 (Klopidogrel) | 5 | 3 |
| B01AC06 (Acetylsalicylic acid) | 88 | 232 |
| B01AE07 (Dabigatranetexilat) | 2 | 7 |
| B01AF01 (Rivaroxaban) | 1 | 7 |
| B01AF02 (Apixaban) | 1 | 0 |
| B01AC07 (Dipyridamol) | 0 | 2 |
| B01AA04 (Fenprokumon) | 0 | 2 |
| B01AX05 (Fondaparinux) | 0 | 1 |
| B01AE05 (Ximelagatran) | 0 | 2 |

PVC = premature ventricular complex.

Figure S1.

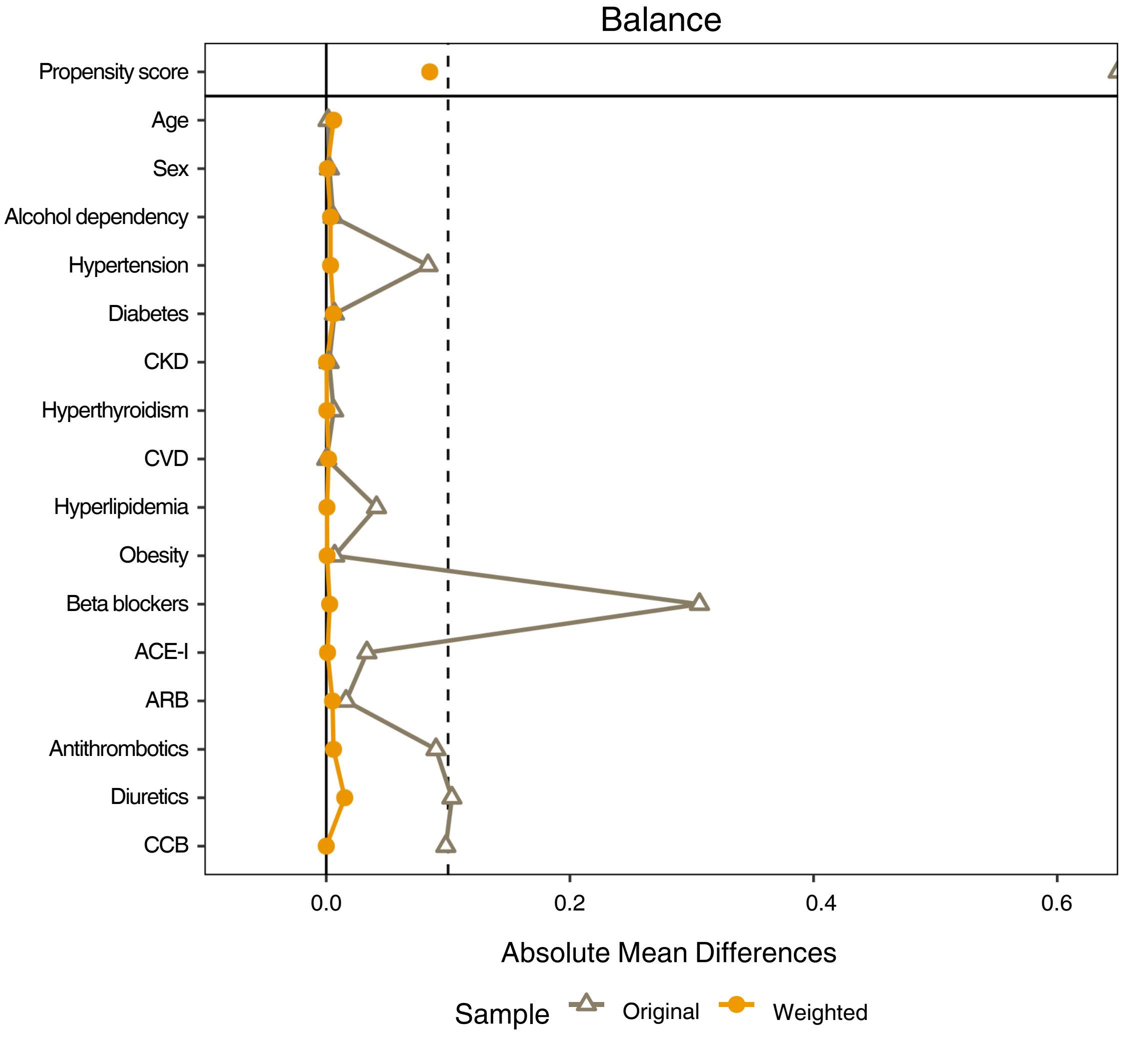


**Figure S1.** Balance of baseline characteristics before and after inverse probability weighting (IPW). CKD = chronic kidney disease, CVD = cerebrovascular disease, ACE-I = angiotensin-converting enzyme inhibitors, ARB = angiotensin receptor blockers, CCB = calcium channel blockers.
